# Retraction Note: Anti-proliferative and immunomodulatory potencies of cinnamon oil on Ehrlich ascites carcinoma bearing mice

**DOI:** 10.1038/s41598-025-94143-6

**Published:** 2025-03-19

**Authors:** Dalia S. Morsi, Sobhy Hassab El-Nabi, Mona A. Elmaghraby, Ola A. Abu Ali, Eman Fayad, Shaden A. M. Khalifa, Hesham R. El-Seedi, Islam M. El-Garawani

**Affiliations:** 1 Zoology Department, Faculty of Science, Menoufia University, Shebin El Kom, 32511 Egypt; 2 Department of Chemistry, College of Science, Taif University, P.O. Box 11099, Taif, 21944 Saudi Arabia; 3 Department of Biotechnology, Faculty of Sciences, Taif University, P.O. Box 11099, Taif, 21944 Saudi Arabia; 4 Department of Molecular Biosciences, Wenner-Gren Institute, Stockholm University, S-106 91 Stockholm, Sweden; 5 Pharmacognosy Group, Biomedical Centre, Department of Pharmaceutical Biosciences, Uppsala University, P.O. Box 591, 752 24 Uppsala, SE Sweden; 6 International Research Center for Food Nutrition and Safety, Jiangsu University, Zhenjiang, 212013 China; 7 Department of Chemistry, Faculty of Science, Menoufia University, Shebin El-Kom, 32512 Egypt; 8 International Joint Research Laboratory of Intelligent Agriculture and Agri-products Processing, Jiangsu Education Department, Jiangsu University, Zhenjiang, 212013 China

Retraction of: *Scientific Reports* 10.1038/s41598-022-14770-1, published online 12 July 2022

The Editors have retracted this Article.

After publication, concerns were raised regarding the data presented in Table 4 and Figure 11. Specifically:

• The standard deviations of several physical properties presented in Table 4, which correspond to animals subject to different experimental conditions, are similar;
• Panels EAC and EAC+Cis in Figure 11 appear to overlap. These Panels represent tissues taken from animals subject to different experimental conditions.

The Authors provided some raw data on request from the Editors. However, the raw data supplied was incomplete, and contained further concerning elements including data that appear in the paper but that appears to be described differently. The Editors therefore no longer have confidence in the results and conclusions presented.

Dalia S. Morsi, Shaden A. M. Khalifa, Hesham R. El-Seedi, and Islam M. El-Garawani did not agree with the retraction. Eman Fayad did not explicitly say whether they agreed or disagreed with the retraction. All other authors did not respond to correspondence from the Editor about the retraction.

